# RETRACTED: Barbillon, G. Plasmonic Nanostructures Prepared by Soft UV Nanoimprint Lithography and Their Application in Biological Sensing. *Micromachines* 2012, *3*, 21–27

**DOI:** 10.3390/mi14051000

**Published:** 2023-05-04

**Authors:** Grégory Barbillon

**Affiliations:** Laboratoire Charles Fabry de l’Institut d’Optique, CNRS UMR 8501, Université Paris-Sud, Campus Polytechnique, RD 128, CEDEX, 91127 Palaiseau, France; gregory.barbillon@laposte.net

The journal *Micromachines* retracts article [1] due to data errors. Following publication, the author contacted the Editorial Office regarding an error in the data. During a review of the data, the author found that the dimensions of the plasmonic nanostructures displayed in Figure 3b (scanning electron microscopy (SEM) image) were not consistent. Indeed, Figure 3b presented plasmonic structures with a diameter of 165 nm and a periodicity of 500 nm, instead of a diameter of 65 nm and a periodicity of 180 nm. Thus, the scale bar of Figure 3b does not correspond to the SEM image of the latter. Consequently, Figure 3b does not correspond to the smoothed extinction spectra displayed in Figure 4, obtained with the plasmonic nanostructures whose dimensions are correct (diameter = 65 nm and periodicity = 180 nm). The impact on the final results is the mismatch or incoherence between the dimensions presented in Figure 3b and the smoothed extinction spectra recorded for the plasmonic nanostructures with correct dimensions (diameter = 65 nm and periodicity = 180 nm), depicted in Figure 4. Adhering to our complaint’s procedure, an investigation was conducted by the Editorial Office and the Editorial Board confirmed the error reported by the author. Thus, article [1] will be retracted.

This retraction was approved by the Editor-in-Chief of the journal *Micromachines*. 

The author agreed to this retraction. 
